# Optimization of the Production Process of Clinical-Grade Human Salivary Gland Organoid-Derived Cell Therapy for the Treatment of Radiation-Induced Xerostomia in Head and Neck Cancer

**DOI:** 10.3390/pharmaceutics16030435

**Published:** 2024-03-21

**Authors:** Jacoba van Zanten, Annelies Jorritsma-Smit, Hans Westra, Mirjam Baanstra, Anne de Bruin-Jellema, Derk Allersma, Bahez Gareb, Rob P. Coppes

**Affiliations:** 1 Department of Clinical Pharmacy and Pharmacology, University Medical Center Groningen (UMCG), 9713 GZ Groningen, The Netherlandsh.h.westra@umcg.nl (H.W.); b.gareb01@umcg.nl (B.G.); 2 Department of BioMedical Sciences, University of Groningen (RUG) and University Medical Center Groningen (UMCG), 9713 GZ Groningen, The Netherlands; m.baanstra@umcg.nl (M.B.); a.l.jellema@umcg.nl (A.d.B.-J.); 3 Department of Radiation Oncology, University of Groningen (RUG) and University Medical Center Groningen (UMCG), 9713 GZ Groningen, The Netherlands

**Keywords:** hyposalivation, human salivary gland stem cells, cell therapy, organoids, ATMP, GMP

## Abstract

Head and neck cancer is a common cancer worldwide. Radiotherapy has an essential role in the treatment of head and neck cancers. After irradiation, early effects of reduced saliva flow and hampered water secretion are seen, along with cell loss and a decline in amylase production. Currently, there is no curative treatment for radiation-induced hyposalivation/xerostomia. This study aimed to develop and optimize a validated manufacturing process for salivary gland organoid cells containing stem/progenitor cells using salivary gland patient biopsies as a starting material. The manufacturing process should comply with GMP requirements to ensure clinical applicability. A laboratory-scale process was further developed into a good manufacturing practice (GMP) process. Clinical-grade batches complying with set acceptance and stability criteria were manufactured. The results showed that the manufactured salivary gland-derived cells were able to self-renew, differentiate, and show functionality. This study describes the optimization of an innovative and promising novel cell-based therapy.

## 1. Introduction

Head and neck cancer is the seventh most common cancer worldwide [1] and is typically diagnosed in older patients associated with heavy use of tobacco and alcohol. Moreover, a rise in the incidence of oropharyngeal cancers is seen due to increasing rates of human papillomavirus (HPV) infection. The effectiveness of prophylactic HPV vaccination is less well defined for oropharyngeal cancer than for cervical cancers [2].

The treatment of head and neck cancers depends on various factors such as the tumor’s severity, histology, stage, and location, but it generally consists of surgery, radiotherapy, chemotherapy, or a combination thereof [2,3].

In about 40% of the patients receiving radiation to the salivary glands during head and neck cancer therapy, hyposalivation is observed due to radiation-induced damage to the salivary gland [4]. This often results in xerostomia (i.e., dry mouth syndrome), a subjective feeling of dry mouth with various symptoms like alterations in speech and taste, difficulties with mastication and swallowing, and an increased risk of developing oral infections and dental caries, speaking, and sleeping problems. Consequently, these symptoms are associated with a significantly reduced quality of life in head and neck cancer patients after treatment [5].

Currently, radiotherapy has an essential role in the management of head and neck cancers.

The salivary glands are exocrine organs whose parenchymal tissue produces and secretes saliva. Due to irradiation, saliva-producing cells, duct cells, blood vessels, and supporting stromal tissue can be affected. [6]. After irradiation, early effects of reduced saliva flow and hampered water secretion are seen, along with cell loss and a decline in amylase production. Based on findings in a rat model, Coppes et al. proposed different phases of radiation-induced loss of salivary gland function [7,8]. The salivary glands secrete saliva in response to various biochemical signals and environmental cues. Saliva contains water, mucus, antibacterial compounds, electrolytes, and various enzymes [9]. Humans have three major salivary glands: the parotid, the submandibular, and the sublingual gland as well as numerous minor glands. The parotid glands are the largest and produce about 45% of daily production. Serous and mucous are the two types of glands that make up the salivary glands. Mucous glands secrete a viscous secretion containing mucin, which is mainly involved in lubrication. The parotid gland only secretes serous saliva. The submandibular glands are much smaller than the parotid glands. Still, they are responsible for about 45% of the total saliva production, are the major contributors to (sero)mucous secretion, and provide about 70% of the unstimulated saliva of the salivary flow during the day [4,10]. The smallest gland is the sublingual gland, which produces about 5% of the total saliva production and mainly secretes mucous saliva [11]. Therefore, prevention of radiation-induced function of the submandibular gland is crucial.

New therapies for radiation-induced xerostomia represent an unmet clinical need. Since the available treatment options for xerostomia generally do not provide adequate management for a permanent cure, the development of new therapies focused on gene therapies, stem cell therapies, and salivary gland bioengineered models has been explored [9,12].

Research has shown that murine salivary gland stem/progenitor cells are capable of in vitro expansion and differentiation into parenchymal acinar- and duct-like cell lineages [13,14]. In addition, the in vivo functionality of murine salivary gland stem/progenitor cells has been demonstrated, showing the potential for new strategies to restore radiation-induced hyposalivation in a mouse model [14,15]. Human salisphere cells, cultured from human salivary gland biopsies, were shown to contain progenitor/stem cells. Moreover, organoid formation with salivary gland structures was observed in an in vitro differentiation assay [15]. The early organoids do not show lobular structures yet and are therefore called spheres.

However, several major challenges are associated with salivary gland stem cell therapy for radiation-induced xerostomia. Salivary gland biopsies should be taken from eligible patients before the initiation of radiotherapy to secure the availability of undamaged salivary gland stem cells. During the patient’s cancer treatment, isolated cells from these biopsies should be stored properly and remain stable for an extended period before further use. Subsequently, the stored cells should be used as the starting material to produce organoids containing a mixture of salivary gland stem, progenitor, and differentiated cells. A salivary gland cell product manufactured from salivary gland biopsies is considered an advanced medicinal therapy product (ATMP), and therefore, the manufacturing process should comply with good manufacturing practice (GMP) guidelines. Due to the unavailability of compendial grade starting and raw materials used during such a manufacturing process, particular attention should be paid to the quality, purity, and safety of these materials to warrant compliance with regulatory guidelines. Finally, the salivary gland organoid cell therapy should be safe and effective. The administered salivary gland cells should differentiate into salivary gland tissue within a given period. Thus, methods to evaluate the efficacy and safety of this novel therapy should be in place.

This study aimed to develop and optimize a validated manufacturing process for salivary gland immature organoid cells containing stem/progenitor cells using salivary gland patient biopsies as a starting material. The manufacturing process should comply with GMP requirements to ensure clinical applicability. The developed salivary gland organoid-derived cell product is intended to treat patients diagnosed with radiation-induced xerostomia. First, a laboratory-scale manufacturing process [16] was further developed to warrant the technology transfer in a GMP setting. Subsequently, the technology transfer of the developed process was performed and further optimized in the GMP setting. To the best of our knowledge, the present study describes for the first time a validated manufacturing process of salivary gland-derived progenitor/stem cells with the ability of organoid formation, complying with GMP requirements, which is a novel and potential therapy for radiation-induced xerostomia.

## 2. Materials and Methods

### 2.1. Study Design

The manufacturing process was initially developed at the Department of BioMedical Sciences of the University Medical Center Groningen (UMCG), The Netherlands [15,16,17]. Based on these results, the manufacturing process was transferred to the Biotech and ATMP’s GMP facility at the UMCG. The first technology transfer batches were manufactured according to the process and protocols based on the results found during development [16].

First, six technology transfer batches were manufactured in the technology transfer runs 1–6 (TTR 1–TTR 6) using the manufacturing process as defined during the development phase [16]. The cells were cultured for four days during passage 0 (P0), 7–10 days during passage 1 (P1), and harvested after 7–11 days after passage (P2). The culture periods were investigated and optimized based on the results of these six batches. Subsequently, three additional technology transfer batches (TTR 7–TTR 9) were manufactured to further investigate the culturing period. In these runs, the cells were cultured for 7–10 days during both P0 and P1, after which the cells were harvested.

In the first nine technology transfer runs (TTR 1–TTR 9), gentamycin was present in the culture medium until the final passage. Although the amount of residual gentamycin was found to be negligible in the final product, gentamycin was omitted from the culture medium during the final passage (P1) to optimize the production process further as well as facilitate the sterility test of the final drug product. Therefore, two additional technology transfer batches (TTR 10 and TTR 11) were produced to investigate the effect of omitting gentamycin during the final step of the culture. Both batches were produced according to the optimized manufacturing processes, with a culture period of 7 days for P0 and 10 days for P1. See an overview in Table 1.

In-process controls (IPCs) were performed at several points during the manufacturing process. The IPCs and acceptance criteria are listed in Table 2, whereas the release tests performed on the final drug product are listed in Table 3. The number of 5 × 10^4^–5 × 10^6^ cells needed for transplantation is based on the results found in mouse studies. Here, between 100 and 10,000 cells were used [14], which proved sufficient for (partial) recovery of the salivary gland function.

Due to the small number of cells in the final drug product, only a limited set of release tests could be performed. Therefore, an extensive IPC testing strategy was chosen (Table 2) to ensure the quality of the intermediate and final drug product (Table 3).

The salivary gland-derived cells are cultured with Matrigel, a 3D matrix. Matrigel itself contains, among other factors, laminin and collagen IV, and the medium contains several growth factors that enhance organoid culture. The salispheres cultured from the salivary gland contain cells with self-renewal and differentiation capacity. An in vitro differentiation assay was performed to assess the potential of human organoid cells to generate functionally mature salivary gland cell lineages. Cultures were evaluated for the formation of mature organoids with salivary gland structures, including branching and the formation of lobular structures. In addition, the expression of cytokeratin (a ductal cell marker), alpha-amylase (a saliva enzyme), and aquaporin-5 (a water channel protein expressed in the apical membrane of acinar cells) was investigated.

Due to the time restrictions for drug release and the small volume of the final drug product, the options for release testing are limited. Therefore, characterization tests were performed on the cultured cells to obtain information on the biological activity and chromosomal stability of the final drug product. The different tests performed to assess the biological activity are the self-renewal potency and a differentiation assay.

### 2.2. Quality Control Starting Material

The tissue was obtained during an elective head and neck dissection procedure for the removal of squamous cell carcinoma of the oral cavity at the UMCG. The patient was screened for Human Immunodeficiency Virus type 1 and 2, Hepatitis B virus, Hepatitis C virus, and Treponema pallidum. Only tissue was used from patients who tested negative for these viruses. Histopathological analysis of the salivary gland was conducted for the detection of malignant cells.

### 2.3. Quality Control Critical Materials

Critical materials used during the production process, such as packaging material, disposable plastics, and raw and starting materials, were evaluated and released based on a formal risk analysis. This ensured the quality of the final drug product since it was not dependent only on the origin of the cells but also on the (biological) properties of the used materials in the entire production process. The risk analyses and considerations were based on the European Pharmacopoeia (Ph. Eur.) 5.2.12 monograph [18]. Not all raw materials used during production were available in compendial grade. The objective of the risk analyses for the critical materials was to ensure the use of pure, high-quality, and safe materials. The materials were classified and the risks assessed based on the intended purpose of the material during the production process, the quality system of the manufacturer of the material, the production process according to GMP principles, the availability of documents (i.e., a certificate of analysis (CoA), a certificate of conformance (CoC), a certificate of origin (CoO), a TSE/BSE statement, and viral safety documents), the control strategy of the manufacturer, and the material specifications. Materials were bought from qualified suppliers. Specifications for critical materials were documented in a material specification form. The quality of plastic disposables was preferably according to USP class VI, and the materials used should be sterile and non-toxic for cells.

### 2.4. Tissue Collection

For the production of non-clinical cultures, non-malignant, human submandibular salivary gland tissue was obtained from donors after informed consent and approval by the Institutional Research Board (IRB) approval. The donors suffered from a squamous cell carcinoma of the oral cavity, in which a head and neck dissection procedure was performed. The submandibular salivary gland was exposed during this procedure and removed as part of the dissection procedure.

The biopsy was taken from the submandibular gland of the patient. The tissue was collected in a 50 mL tube containing Hank’s Balanced Salt Solution (HBSS no Ca/Mg/Ph red; ThermoFisher Scientific, Waltham, MA, USA) with 1% Human Serum Albumin (HSA; Prothya Biosolutions, Amsterdam, The Netherlands) and 50 µg/mL Gentamycin (Brocacef, Maarssen, The Netherlands) and placed on ice during transport.

### 2.5. Dissociation of Tissue and Generation of Cell Clumps

A maximum of 2 g of tissue was used for digestion. The tissue was divided over one or more tubes using an automated tissue gentleMACS dissociator (Miltenyi Biotec, Bergisch Gladbach, Germany) with 100 mg of tissue per tube. The tubes contained 6 mL HBSS medium supplemented with 1% HSA, gentamycin (50 µg/mL), Collagenase NB6 (12.5 U/mL; Nordmark Pharma, Uetersen, Germany), Pulmozyme (Roche, Woerden, The Netherlands), and 6.3 mM CaCl2. Following tissue dissociation, the samples were incubated in a water bath at 37 °C under gentle shaking. After 30 min of incubation, an additional dissociation program run was performed, and the samples were returned to the water bath for another 30 min at 37 °C under gentle shaking. Next, a centrifugation step was performed (5 min at 400× *g*). Subsequently, cells were washed twice with HBSS medium and passed over a 100 µM filter. The cells were then resuspended in HBSS medium. The cell clumps and cells were counted with a Scepter™ (Millipore, Amsterdam, The Netherlands) handheld cytometer, and a small aliquot was taken to examine the presence of cell clumps under a microscope.

### 2.6. Freezing and Storage of Isolated Cells

The intermediate cell product was frozen in a 1 mL Cryostor CS-10 freezing medium (Stemcell Technologies, Vancouver, CO, Canada) supplemented with 10 µM Rho-kinase inhibitor Y-27632 (Abcam, Cambridge, UK). In each vial, 5–10 × 10^6^ cells were added for storage. Cells were frozen using a Kryo 560 planer (Planer Ltd., Middlesex, UK) and transferred to a liquid nitrogen container, where they were stored until further use.

### 2.7. Salisphere Cell Culture

The cells of one vial were thawed by gently shaking the vial in a 37 °C water bath till a small ice clump remained. One mL of DMEM:Ham’s F12 medium (Thermo Fisher Scientific, Waltham, MA, USA) supplemented with 1% HSA was added dropwise to the vial, and cells were subsequently transferred to a tube containing medium with 1% HSA. After centrifugation (5 min at 400× *g*), cells were resuspended in a culture medium. The culture medium consisted of DMEM:Ham’s F12 medium supplemented with 50 µg/mL gentamycin and 2 mM Glutamax CTS (Thermo Fisher Scientific, Waltham, MA, USA) with the following growth factors: 40 ng/mL EGF (Miltenyi Biotec, Bergisch Gladbach, Germany), 20 ng/mL FGF2 (GMP grade, Peprotech Hamburg, Germany), 1× CTS N2 (Thermo Fisher Scientific, Waltham, MA, USA), 10 µg/mL insulin (SAFC Merck Group, Amsterdam, The Netherlands), 1 µM dexamethason (Fagron, Capelle a/d IJssel, The Netherlands), 100 ng/mL R-spondin-1 (SAFC Merck group, Amsterdam, The Netherlands), 10 µM Rho-kinase inhibitor Y-27632, and 1 µM TGFβ-inhibitor (Biotechne-Tocris, Abingdon, UK). The cells were counted as spheres with the scepter and plated in Matrigel basement membrane matrix (Corning Life Sciences, Corning, NY, USA). A density of 0.4 × 10^6^ cells/well was plated in Matrigel in a 12-well plate. To each well, 1 mL culture medium was added, and the cells were cultured as passage 0 (P0).

### 2.8. Cell Culture and Generation of Spheres

For the following passage (P1), spheres were detached from the Matrigel by adding neutral protease (1 U/mL; Nordmark Pharma GmbH, Uetersen, Germany) for 1 h at 37 °C. All spheres larger than 5.2 µm (0.5 bars × 10 × ocular) were counted. Next, spheres were dissociated into single cells by adding GMP-grade and recombinant trypsin (Roche Diagnostics, Penzberg, Germany). The trypsin was deactivated by adding DPBS (Thermo Fisher Scientific, Waltham, MA, USA) supplemented with 0.2% HSA. Single cells were counted, and viability was measured by trypan blue staining. The single cells were suspended in a culture medium with or without gentamycin and passaged in Matrigel. A density of 0.2 × 10^5^ cells/well was plated in Matrigel in a 12-well plate. To each well, 1 mL culture medium was added, and the cells were cultured for 7-10 days in a humidified CO_2_ incubator (5% CO_2_, 37 °C).

### 2.9. Washing and Formulation of the Final Cell Product

At the end of P1, single cells were generated as described in the section “Cell culture and generation of spheres”. Cells were centrifuged for 10 min at 400× *g* and resuspended in PBS with 0.2% HSA. Cells were again centrifuged for 5 min at 400× *g* and subsequently resuspended in the final formulation buffer, which consisted of phosphate-buffered saline (PBS; 10 mM with 148 mM sodium chloride) set at pH = 7.0 (EP0473, Apotheek A15, Gorinchem, The Netherlands) supplemented with 1% HSA.

### 2.10. Number of Spheres

The determination of the number of spheres was performed by microscopy. A small aliquot of the culture was taken, and the spheres were examined under the microscope. All spheres larger than 50 µm were counted using the reticule.

Number of Single Cells and Viability

The single-cell suspension was tested for cell viability and cell density with the trypan blue assay. The cells were counted under a microscope using a Bürker–Türk counting chamber, counting both viable (colorless) and dead (blue) cells.

### 2.11. Sphere-Forming Potency

To determine stemness, the self-renewal potency of the cells was determined by measuring the sphere-forming potency in the following passage. Primary spheres were examined under the microscope, and the number of spheres was counted using the reticule. First, spheres were detached from the Matrigel by adding neutral protease (1 mg/mL) for 1 h at 37 °C. All spheres larger than 5.2 µm (0.5 bars × 10x ocular) were counted. The sphere-forming potency (SFP) was determined using the following formula:

SFP = (Number of spheres/seeded cells) × 100%

### 2.12. Differentiation Assay

An in vitro differentiation assay was performed to assess the potential of human salisphere cells to generate functionally mature salivary gland cell lineages. Cultures were evaluated for the formation of organoids with salivary gland structures, including branching and the formation of lobular structures.

Salispheres were kept in culture in a research laboratory after P1 in the following passage, P2, for 7 days in an expansion medium containing Wnt3a, R-Spondin, and noggin. The medium was harvested, and salispheres were released from the Matrigel. They were harvested and spun at 400 g for 5 min. The pellet was resuspended in 1 mL differentiation medium and kept on ice. The salispheres were counted and seeded at a final density of 20–30 per 75 µL of gel (25 µL medium + 50 µL Matrigel) in a Matrigel pre-coated 96-well plate. Following polymerization of the gel at 27 °C, 150 µL of a differentiation medium was added on the top of the gel. The salispheres were harvested after 30 days for immunohistochemistry analysis of salivary gland-specific proteins. The differentiation medium consisted of DMEM/F12, pen/strep, glutamax (Thermo Fisher Scientific, Waltham, MA, USA), 50 ng/mL HGF, 40 ng/mL FGF-10 (PeproTech, London, UK), 100 ng/mL heparin salt (Stemcell Technologies, Vancouver, BC, Canada), 1 µM DAPT, 100 ng/mL Carbachol (SAFC Merck Group, Amsterdam, The Netherlands), and 10% FCS (Greiner Bio-One, Kremsmünster, Austria). Cultures were evaluated for the formation of salivary gland structures, including branching and lobular structures.

### 2.13. Bioburden

Bioburden was determined according to Ph. Eur. 2.6.12. The filtration method was used to remove gentamycin, which was used as a medium supplement in the initial steps of the process. The test was performed on a sample of the intermediate product on the day of cell isolation from the tissue.

### 2.14. Sterility

Sterility was determined using the BACTEC culture system (Beckton Dickson, NJ, USA), a combination of an automated culture system and detection based on CO_2_ production. The test was performed as described in Ph. Eur. monographs 2.6.27 and 5.1.6. The BACTEC Type Peds Plus/F culture vials contain non-ionic adsorbing and cationic exchange resins, which inactivate antibiotics. Culture vials were inoculated with a 1 mL sample and cultured for 5 days while CO_2_ production was continuously monitored. The growth-promoting capacity of the BACTEC test media was confirmed by a validation study.

Bacterial Endotoxins

The determination of bacterial endotoxins was performed using the Limulus amebocyte lysate (LAL) test as described in Ph. Eur. 2.6.14.

### 2.15. Histochemical Staining

In addition to the differentiation test, the expression of cytokeratin (a ductal cell marker), alpha-amylase (a saliva enzyme), and aquaporin-5 (a water channel protein expressed in the apical membrane of acinar cells) was investigated.

Duct-like structures from the differentiation assay were embedded in HistoGel (Thermo Fisher Scientific, Waltham, MA, USA) and paraffin. Material from the GMP technology transfer batches TTR 7-9 was subjected to the following histochemical staining methods:

Alcian blue: staining of all acidic mucins. Staining is performed for 30 min with a 1% solution, pH 2.5.

PAS staining: neutral mucins but also acid mucins that contain silica acid. Staining is performed by 5 min incubation in periodic acid (0.5%) and 15 min in Schiff’s base solution.

Aquaporin 5 staining: the first monoclonal antibody AQP5 (Abcam Ltd., Cambridge, UK) is used that recognizes water channels. The secondary antibody used is Goat αRabbit IgG, Alexa Fluor 488 (ThermoFisher Scientific, Waltham, MA, USA).

Amylase staining: antibody-based staining for the detection of amylase is performed with Anti-α-Amylase (SAFC, Merck group, Amsterdam, The Netherlands) and Goat αRabbit IgG, Alexa Fluor 488.

CK19: staining for cytoskeleton marker (mature duct) is performed with CK19 antibody (Abcam Ltd., Cambridge, UK) and secondary antibody Goat αRabbit IgG, Alexa Fluor 488.

### 2.16. Gentamycin Detection

Gentamycin was measured using a particle-enhanced turbidimetric inhibition immuno-assay (PETINIA). This assay was based on competition between gentamycin in the sample and microparticles coated with gentamycin. The gentamycin-coated microparticles will agglutinate in the presence of a gentamycin antibody. The agglutination will be prevented in the presence of gentamycin in the sample. Agglutination will be measured photometrically using the ARCHITECT immunoassay system (Abbot, IL, USA), and the amount of gentamycin present in the sample can be calculated based on the change in absorption.

### 2.17. Stability Study

The cellular material from batches TTR 10 and TTR 11 manufactured with the optimized manufacturing process under GMP conditions was used to investigate the stability of the final drug product. The stability was investigated with cells in the formulation buffer at a concentration of 1 × 10^6^ cells/mL and stored in the primary container (glass vials). Two vials per batch were prepared with a volume of 1–1.5 mL cell suspension each. One vial was stored at a temperature of 5 ± 3 °C, and the second vial was stored at ambient room temperature (RT). Testing was performed at 2 h, 4 h, and 5 h after filling. The cell suspension was tested for viability, cell yield compared to T = 0 (time of filling), and the presence of spheres. To investigate the sphere-forming capacity, the cells of the stability samples were returned to the culture medium and evaluated after one week. The release and end-of-shelf-life specifications of the stability study are given in Table 4.

### 2.18. Aseptic Process Validation

Validation of aseptic processes should include a process simulation test according to the GMP guidelines for the production of sterile medicinal products (Annex 1). Therefore, the entire manufacturing process was simulated with the sterile microbiological growth medium tryptic soy broth (TSB; Biotrading, Mijdrecht, The Netherlands). Three consecutive simulation tests with the microbiological growth medium were performed, in which all critical process steps were simulated. The isolation of the cells from the tissue, the culture steps P0 and P1, and the harvest of cells were mimicked. Three vials containing 1.0–1.5 mL of TSB were stored in the closed primary container (glass vial). In addition, eight intermediate samples were stored in 50 mL tubes. All samples were incubated for 7 days at 22.5 °C and immediately after for 7 days at 32.5 °C. After incubation, a growth promotion test according to Ph. Eur. 2.6.1 and 2.6.12 was conducted on the vials (Eurofins Bactimm, Nijmegen, The Netherlands). For the incubation period, the acceptance criterion was no microbial growth. The acceptance criterion for the growth promotion test was “positive growth”, as stated in the respective Ph. Eur. monographs.

## 3. Results

### 3.1. Raw Materials

For each raw material used in the production process, a material dossier was compiled. The material dossier included information about the manufacturer and the production process of the raw material, copies of the available documents (e.g., CoA, CoO, and CoC), and a risk analysis focused on potential risks regarding microbiological contamination, viral safety, TSE/BSE, and toxicity. The release criteria of each raw material were determined based on all of the available information.

The materials of animal or human origin that came into direct contact with the cellular product are listed in Table 5. For material of animal or human origin, an adventitious agent safety evaluation was performed based on the species and origin of the material.

The used N2 CTS contains transferrin derived from human plasma. The source of human plasma complied with the guidelines on plasma-derived products and was FDA-approved. Recombinant human R-Spondin-1 was derived from a master cell bank (MCB) produced in a medium containing animal-derived materials. For the production of the protein itself, no animal-derived materials were used. The product’s safety was considered warranted since, between the culture of the MCB and the final product, three different and sequential purification processes were performed and the MCB was tested for the absence of cytopathological and bovine viruses.

Matrigel was a raw material designated with a qualification of “possible risk” because it was animal-derived. The manufacturer mitigated the risk by testing for microbial contamination (sterility, mycoplasma, and endotoxin), mycoplasma, and the absence of several viruses.

The results from the risk assessments for the raw materials used showed that the materials were suitable for production, according to the GMP conditions of the salivary gland stem cells.

### 3.2. GMP Technology Transfer and Process Optimization

The cells isolated from the biopsies of salivary glands and used for the culture were derived from seven different donors. As shown in Table 6, the isolated cells were used for one, two, or three TTRs.

The results of the isolation procedure from the tissues and the specifications are shown in Table 7. The mass of the biopsy ranged from 0.5 to 2.4 g. Although the mass of biopsies from isolations 2 and 5 was below the target range, the tissue was used for further processing. The results showed that the number of cells isolated from the biopsy did not correlate with the size of it. Based on our experience, the cell yield from the biopsy of the submandibular salivary gland also depends on the location where the biopsy is taken from the gland and on the fat percentage of the tissue. This confirms the findings found with the parotid gland [19].

**Figure 1:**
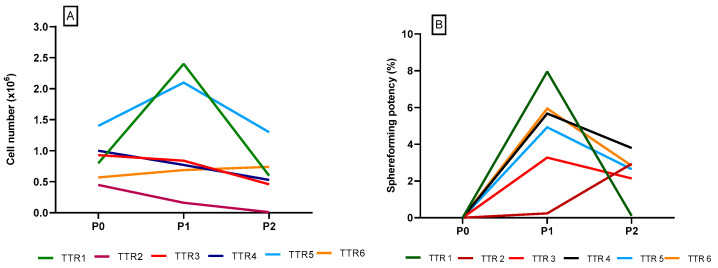
(**A**): The number of cells after P0, P1, and P2 in the technology transfer batches TTR1-6. (**B**): The sphere-forming potency of the technology transfer batches TTR 1–TTR 6.

The first six batches (TTR 1–TTR 6) were manufactured using the manufacturing process defined during the development phase. Here, cells were cultured for 4 days during P0, 7–10 days during P1, and harvested after 7–11 days after P2. The batch specifications and test results are shown in Table 8. The number of cells found after culture in P0, P1, and P2 are shown in Figure 1.

In the batches TTR 1–TTR 6, the cells were cultured for 4 days in P0. The results showed that the increase in cell amount was relatively low. In addition, the cultures showed a decrease in cell number in P2.

The sphere-forming potency was regarded as the capacity for self-renewal and was determined for each TTR 1–TTR 6. The sphere-forming potency was calculated as a percentage of the number of spheres divided by the number of seeded cells. The results from these batches showed that the sphere-forming potency increased in P1 but declined in P2 (Figure 1B).

It was hypothesized that the culture period of P0, in which the cells also need to recover from the freeze-thaw cycle, was too short. Therefore, the manufacturing process was adjusted with respect to the length of the culture period. Moreover, since no significant expansion of cells could be seen in P2, the number of passages was reduced. The culture time of P0 was extended from 4 to 7–10 days, and the cells were harvested after 7–10 days in P1. Subsequently, an additional three technology transfer batches (TTR 7–TTR 9) were manufactured with this further optimized manufacturing process to investigate the effects of the adjustments. The specifications and test results of these batches are shown in Table 9.

**Figure 2:**
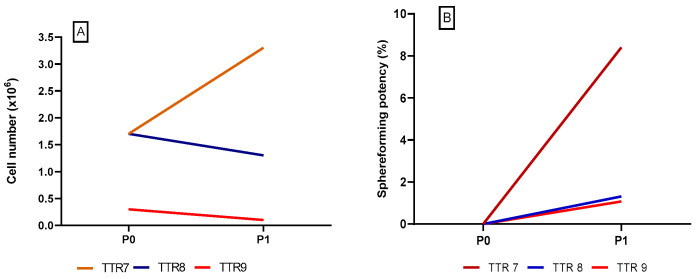
(**A**): The number of cells after P0 and P1 in the technology transfer batches TTR 7–TTR 9. (**B**): The sphere-forming potency of the technology transfer batches TTR 7–TTR 9.

Figure 2 shows the number of cells and sphere-forming potency of the TTR 7–TTR 9 batches, respectively. The sphere-forming potency of these batches was comparable to the batches TTR 1–TTR 6 and showed an increase from P0 to P1. The results show moderate cell and sphere numbers despite the adjustments to the manufacturing process. However, the adjustment did result in a shorter manufacturing time due to the omission of P2, which was considered a process optimization step.

In the batches TTR 1–TTR 9, gentamycin was present in the culture medium up until the final passage. Although the residual gentamycin was negligible in the final drug product, it was decided to omit gentamycin from the culture medium during the final passage. This is to optimize the production process further and to facilitate the sterility test of the final drug product.

The technology transfer batches TTR 10 and TTR 11 were manufactured to investigate the effect of omitting gentamycin during the final step of the culture. The impact of omitting gentamycin in P1 was investigated by analyzing the sphere-forming potency and population doublings. Both batches were produced according to the further optimized manufacturing processes, with a culture period of 7 days for P0 and 10 days for P1.

**Figure 3:**
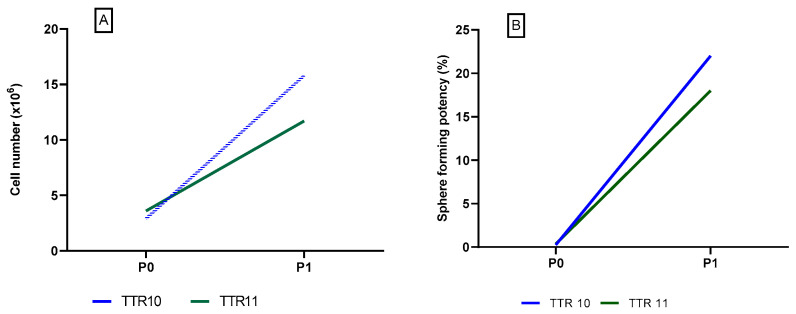
(**A**): The number of cells after P0 and P1 in the technology transfer batches TTR 10–TTR 11. (**B**): The sphere-forming potency of the technology transfer batches TTR 10–TTR 11.

The specifications and test results of the batches TTR 10–TTR 11 are shown in Table 10, and the number of cells and sphere-forming potency of the cells are shown in Figure 3.

The results showed a significantly higher cell yield and a higher percentage of sphere-forming potency for the technology transfer batches TTR 10–TTR 11 compared to the former batches TTR 1–TTR 9.

Importantly, omitting gentamycin made it possible to perform a sterility test on the final drug product. Furthermore, omitting gentamycin was also desired because of patient safety since residual antibiotics in the final drug product were considered a safety risk due to the sensibilitating nature of the compound. Therefore, the final manufacturing process with which the final batches were manufactured was optimized for producing human salivary gland stem cells under GMP conditions.

The final drug product will be parametrically released based on the results of the sterility and bacterial endotoxin test results on product samples taken 3 days before administration (Table 2 and Table 3). The results of the sterility testing on the day of administration will be known after the cells have been transplanted into the patient.

### 3.3. Recovery after the Storage Period

After cryopreservation and thawing, the recovery of the cells after storage in the gas phase of liquid nitrogen was determined. The recovery of the cells after the cryopreservation was 57–123%, with a mean of 93% and a median of 94%. The period between cryopreservation and thawing of the cells varied from 5 to 38 weeks, with a mean of 20 weeks and a median of 15 weeks. The results showed that the recovery of the cells was not influenced by the storage time in the gas phase of liquid nitrogen. The recovery, growth, and viability of the cells were not donor-dependent. Therefore, the results show that the cells were stable for up to 38 weeks in the gas phase of liquid nitrogen.

### 3.4. Formulation and Fill

The salivary gland stem cells were formulated into the cellular drug product on the day of administration. The formulation buffer consisted of a 10 mM sodium phosphate and 148 mM sodium chloride buffer solution set at pH 7.0 supplemented with 1% HSA. All buffer components were of compendial grade (Ph. Eur.), which ensured the clinical applicability of the formulation buffer.

The cells were stored until administration in a 2 mL glass vial in a concentration range of 5 × 10^4^–5 × 10^6^ cells/mL.

### 3.5. Potency Test

An in vitro differentiation assay was performed to assess the potential of human salisphere cells to generate functionally mature salivary gland-derived organoids. Cultures were evaluated for the formation of organoids with salivary gland structures, including branching and the formation of lobular structures. In addition, the expression of cytokeratin (a ductal cell marker), alpha-amylase (a saliva enzyme), and aquaporin-5 (a water channel protein expressed in the apical membrane of acinar cells) was investigated.

**Figure 4:**
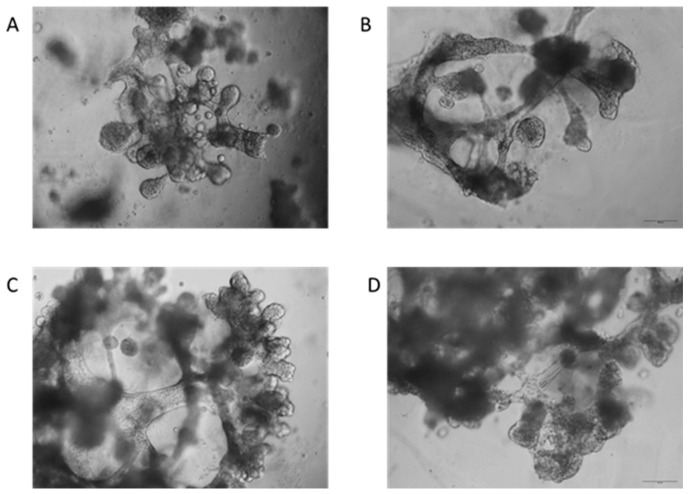
Representative images of the differentiated cells forming duct-like structures. Single cell-derived salispheres derived from P1 cultures of technology transfer batches TTR 7 (**A**,**B**) or TTR 8 (**C**,**D**) were differentiated in the gel for up to three weeks. Phase contrast microscopy pictures were taken at the end of the culture period. The images show the formation of organoids with salivary gland structures, including branching and the formation of lobular structures (scale bars in images (**B** ) and (**D**) 500 µm).

Differentiation potency was tested for all technology transfer batches (TTR 1–TTR 11). Differentiation was found in all the investigated cultures, regardless of the observed poor or good growth. Moreover, differentiation was not limited to a specific passage and was observed after all passages P0, P1, and P2. Figure 4 shows representative images of the differentiated cells forming duct-like structures.

The duct-like structures from the differentiation assay were embedded in HistoGel and paraffin. The cells from the technology transfer batches TTR7-9 were subjected to histochemical staining: Alcian blue, PAS staining, Aquaporin 5 staining, amylase staining, and staining for the cytoskeleton marker CK19 (mature duct).

**Figure 5:**
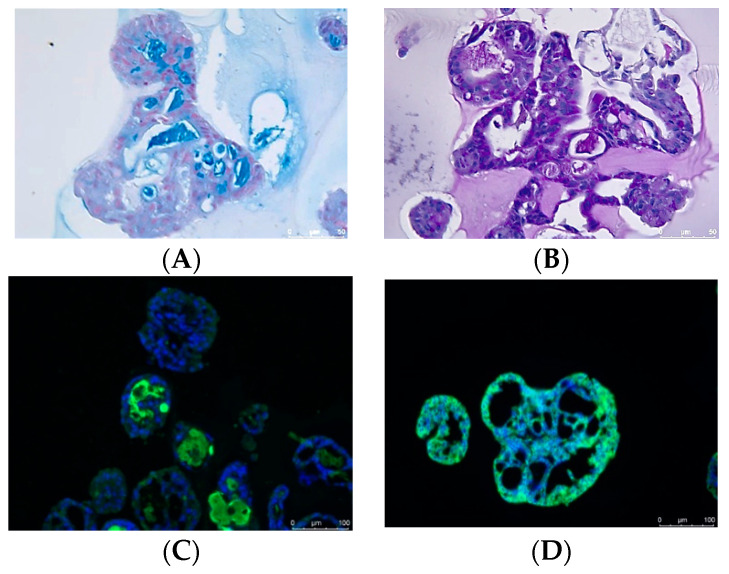
Representative images of the staining of the duct-like structures. (**A**): Alcian blue staining. (**B**): PAS staining. (**C**): Amylase staining. (**D**): CK19 staining.

The results of the histochemical staining are shown in Table 11. The results show that in the tissue sections of all three batches, positive staining was seen for CK19, showing that mature ducts were present. Positive staining was also observed for PAS, Alcian blue, and amylase detection in most tissues. Representative images of the histochemical staining are shown in Figure 5. These results demonstrate that salivary gland stem cells, generated via either the initial or optimized manufacturing process, can differentiate into both acinar and ductal lineages and express mature salivary gland functional proteins such as mucin and amylase.

### 3.6. Stability Study

The cellular material from the technology transfer batches TTR 10–TTR 11 was used to investigate the stability of the final drug product. Table 12 shows the stability data of the final drug product stored either at room temperature or refrigerated (5 ± 3 °C).

The viability proved to be the most critical parameter (Table 12).

The results of the samples taken at T = 4 or T = 5 h show that some samples stored at room temperature did not meet the specification of ≥70% viable cells. The viability results were within specification for the cells stored at 5 ± 3 °C for ≤4 h. Viable cells were returned to a culture medium after storage in the formulation buffer and retained their capacity to form spheres. In addition, the total cell yield after 2, 4, and 5 h was considered sufficient, with both an average and a median of 95% in relation to the number of cells at T = 0. Based on these results, the stability and storage condition of the final drug product were set at 4 h and stored at 5 ± 3 °C, which was considered sufficient for the phase I clinical trial during this stage of development.

### 3.7. Impurities

Before transplantation, the cultured salivary gland stem cells were collected from the culture medium, washed, and resuspended in a formulation buffer. During the process validation, the amounts of residual gentamycin were determined as a model for other impurities and to determine the reduction factor of the washing procedure.

In the technology transfer batches TTR 1–TTR 6, gentamycin was used from the complete culture period until P2. After harvest and washing, the residual amount in the final drug product was below the detection limit for all investigated samples (<0.5 mg/L). Since the amount of gentamycin in the culture medium was 50 mg/L, the reduction factor was ≥100× after the first and presumably even more after the final washing step.

The culture medium also contained other small molecules, such as glutamine and dexamethasone, and larger molecules, such as EGF, FGF, and insulin. Based on the reduction factor as determined for gentamycin, it was assumed that for the other medium components, a reduction factor of ≥100× could be expected since none of these components are likely to specifically bind to the cells, spheres, or other materials used during the entire production process.

### 3.8. Aseptic Process Validation

Three aseptic process validation batches were produced. All three batches complied with the incubation period acceptance criterion of no microbial growth. The batches also complied with the subsequent growth promotion tests. These results show that the manufacturing process of the salivary gland stem cells was considered validated at this stage of development.

## 4. Discussion

The present study describes for the first time the optimization and production process of an innovative and promising novel cell-based therapy for the treatment of radiation-induced xerostomia. The manufactured cells retained the ability to self-renew and differentiate and were shown to be functional, as assessed by the potency tests.

Several significant challenges are associated with salivary gland stem cell therapy in radiation-induced xerostomia. First, salivary gland biopsies should be taken from eligible patients before the initiation of radiotherapy to warrant the availability of undamaged salivary gland stem cells. During treatment of the patient, isolated cells from these biopsies should be appropriately stored and remain stable for an extended period before further use. Subsequently, the stored cells should be used as starting material to produce a mixture of salivary gland stem cells and progenitor cells. The culture technique should result in stable, viable, and potent stem/progenitor cells formulated in a suitable formulation buffer that can be administered to the patient.

In the technology transfer of the production process, the biggest challenge was to optimize the culture conditions using medium components with quality requirements suitable for clinical use. Due to the early phase of clinical development and the small-scale production size of the product, the use of research-grade products could not be avoided. A risk analysis was performed, focusing on potential risks with regard to microbiological contamination, viral safety, TSE/BSE, and toxicity. The release criteria for each raw material were determined based on all available information. Two raw materials of biological origin, Wnt3a and Noggin, were omitted during the development phase since these components did not meet the pre-set requirements for therapeutic use.

Several changes in the technical transfer process were introduced to improve the cellular yield. The culture time after cryopreservation and thawing was extended to 7–10 days. Moreover, P2 was omitted because growth and self-renewal potency did not improve after P1. Due to the extension of the first culture step in which the cells could recover from the storage period, further expansion was seen in P1. The number of cells obtained was relatively low, with a median of 0.8 × 10^6^. However, this was considered a sufficient therapeutic dose during this stage of development.

The antibiotic gentamycin was used to reduce the bioburden due to the procurement of living tissue. Initially, gentamycin was used during the whole culture period, but during process optimization, it was omitted from the culture in P1. This resulted in the absence of gentamycin in the final drug product, facilitating the sterility test. Although only a few batches were manufactured without gentamycin in P1, the results showed that the cell’s quality, viability, and yield improved. These data demonstrate that omitting gentamycin during the production process positively affected the cultured cells. Previous studies [20] have shown that gentamycin has a negative effect on cellular metabolism and could be cytotoxic in higher concentrations. In addition, the salivary gland stem cells were cultured in a serum-free medium. Without serum proteins, the gentamycin concentration might be even more cytotoxic.

Due to the small scale of the product and the short period between harvest and administration, only a few release tests were possible. Product release testing was restricted to cell number, presence of salivary spheres, sterility testing, and appearance. The results showed that the drug product of the technical runs complied with the specifications set for sterility, endotoxin, and viability. The TTR 2 and 9 yields did not meet the target number of 500,000 cells. However, the number of cells in both runs was sufficient for the required number of cells in the vial (i.e., 5 × 10^4^–5 × 10^6^).

In-process testing was more extensive and focused on the cell number, viability of the cells, and the presence of spheres. Critical process parameters and characterization tests were performed during the validation runs to substantiate fewer tests during the release of the drug product.

For instance, potency was an important analytical assay to assess the functionality of the product. The potency test results showed that the cells could differentiate into duct-like structures and were able to secrete saliva and salivary enzymes. All test results complied with the pre-set acceptance criteria. Therefore, the manufacturing process was considered validated during this stage of development.

The production process of the present study is suitable for small-scale early clinical trials. However, the culture process is labor-intensive and requires many open, aseptic handlings. The production process must be further optimized for larger-scale applications. To improve the culture conditions, the availability of clinical grade medium components to increase the salivary gland stem cell potency should be investigated. Another process optimization step could be the replacement of Matrigel with another suitable alternative. The cells need a three-dimensional extracellular micro-environment to facilitate optimal growth conditions. Removing the cells from the Matrigel was easy by using the enzymes collagenase and neutral protease. However, a major disadvantage was that the Matrigel was extracted from the Engelbreth–Holm–Swarm mouse sarcoma. This is not a well-defined, animal-derived matrix consisting of a laminin/collagen IV-rich basement membrane extracellular environment supplemented with a number of unknown growth factors. The use of Matrigel in the phase I clinical study was justified due to the extensive viral as well as microbial and mycoplasma testing. For future research and process optimization, a non-animal-derived and well-defined matrix should be investigated as a suitable alternative to further warrant the safety of used raw materials. Other researchers also investigate different materials, but the in vitro and in vivo performances need to be studied further [9].

The stability data of the final drug product show that the formulation was not stable at room temperature. The stability of the formulation was increased during refrigerated storage, with a resulting shelf life of 4 h, which was considered sufficient for a phase I clinical trial during this stage of development. A limited shelf life is typically observed for cellular products stored at room temperature or in refrigerated conditions [21]. Formulation studies should focus on developing clinical grade stability-enhancing formulation buffers to optimize the final drug product further. An increased drug product stability is desired since it would further facilitate clinical applicability.

It should be realized that, at the moment of transplantation, cells are not yet differentiated. The cell population returned to the patient can contain multiple progenitor populations, which are difficult to define. The mechanism of the differentiation process is currently not well defined and needs to be further investigated. Results from in vitro studies might be different for the in vivo situation.

A functional salivary gland requires complex interactions among several cell types and incorporation in the gland’s microenvironment. Vascularization is essential for the delivery of oxygen and the removal of cellular waste. Mesenchymal stem cells (MSCs) might be an option to improve tissue vascularization. In a systematic review of preclinical studies by Fenger Carlander et al. [22], it was demonstrated that MSCs had a positive effect on salivary gland functioning with a significant increase in the salivary flow rate. A pilot study with IFNγ-stimulated MSCs in the submandibular gland of patients with radiation-induced xerostomia was well tolerated, and some patients experienced an increase in saliva production [23]. Due to the immunoregulatory properties of MSCs, chronic inflammation and fibrosis might be reduced. Taken together, a combined approach of returning autologous salivary gland organoid cells containing stem/progenitor cells in combination with MSCs is an encouraging option for treatment that needs to be further investigated.

The present study describes a validated manufacturing process for an innovative and novel cell-based therapy for treating xerostomia. The data presented substantiates the clinical applicability of the cell therapy in a phase I clinical trial in which the efficacy and safety of the therapy can be assessed. This clinical trial is currently ongoing (Eudra-CT-number NCT04593589).

## Figures and Tables

**Table 1:** Overview of the technology transfer runs.

TTR	P0	P1	Gentamycin
1–6	4 days	7–10 days	P0 and P1
7–9	7–10 days	7–10 days	P0 and P1
10 + 11	7–10 days	7–10 days	P0

**Table 2:** The IPCs performed during the manufacturing process.

Test	Method	Specification
Tissue collection		
Weight of salivary gland tissue	Scale	Target weight ≥ 1 g
Dissociation and generation of cell clumps		
Number of total cells after isolation	Cell counting with scepter	For information
Presence of cell clumps after isolation	Microscope	Present
Bioburden	Ph. Eur. ^1^	<10 CFU/mL
Endotoxin	Ph. Eur.	<10 EU/mL
Thawing of intermediate cell product		
Number of cell clumps	Microscope	For information
Recovery after thawing	Calculation: ratio of cells to the number of cells initially frozen	For information
Cell culture (passage 0)		
Days in culture	n.a. (calculation) ^2^	4–10 days
Presence of spheres before harvest	Microscope	For information
Number of spheres	Microscope	For information
Number of single cells	Cell counting	For information
Viability	Cell counting	For information
Cell culture (passage 1)		
Days in culture	n.a. (calculation) ^2^	7–10 days
Presence of spheres before harvest	Microscope	For information
Number of spheres	Microscope	For information
Number of single cells	Cell counting	For information
Viability	Cell counting	For information
Cell culture (passage 2)		
Days in culture	n.a. (calculation) ^2^	7–11 days
Presence of spheres before harvest	Microscope	For information
Number of spheres	Microscope	For information
Number of single cells	Cell counting	Target: ≥500,000
Viability	Cell counting	≥70%

^1^ method in conformance with the European Pharmacopoeia. ^2^ Method not applicable; number of days calculated.

**Table 3:** The release tests and specifications of the final drug product.

Test	Method	Specification
2 days before administration		
Sterility	BACTEC ^1^	No growth
Endotoxin	Ph. Eur.	<1 EU/mL
Washing and formulation		
Presence of spheres before harvest	Microscope	For information
Number of spheres	Microscope	For information
Number of single cells	Cell counting	Target: ≥500,000 cells
Viability	Cell counting	≥70%
Appearance	Visual inspection	Clear to slightly opalescent solution
Sterility	BACTEC	No growth
Endotoxin	Ph. Eur.	<1 EU/mL
Number of cells in a vial	Calculation from the number of cellular yields	5 × 10^4^–5 × 10^6^

^1^ The BACTEC radiometric method, based on the measurement of CO_2_ produced by bacteria.

**Table 4:** The release and end of shelf-life specifications of the drug product stability study.

Test	Release Specification	End of Shelf Life Specification
Single cell number	5 × 10^4^–5 × 10^6^ cells	Within 20% of release value
Viability	For information	≥70%
Spheres present	Present	Present

**Table 5:** The materials of animal or human origin that came into direct contact with the cellular product.

Material	Origin	Risk
HSA (human serum albumin)	Human	Registered medicinal product, negligible risk
Collagenase NB6	Microbial, but with animal-derived components in the production process	EDQM Certificate of Suitability, negligible risk
Dornase alpha (Pulmozyme)	Animal (produced in Chinese Hamster Ovary cell line)	Registered medicinal product, negligible risk
Matrigel	Mouse (Material extracted from the Engelbreth–Holm–Swarm mouse sarcoma)	Possible risk; mitigated by the supplier’s quality control testing
N2 CTS	Human transferrin from plasma	Negligible risk
Rec human R-spondin-1	Animal-derived material used in the production of the Master Cell Bank (Chinese Hamster Ovary cell line)	Negligible risk

**Table 6:** The source of the donor material used for the 11 different TTRs.

Isolation from Donor	Technical Transfer run (TTR)
Isolation 1	TTR 5
Isolation 2	TTR 3 and TTR 7
Isolation 3	TTR 1 and TTR 4
Isolation 4	TTR 6
Isolation 5	TTR 2, TTR 8, and TTR 9
Isolation 6	TTR 10
Isolation 7	TTR 11

**Table 7:** The specifications and test results of the intermediate cell product (isolation of cells from tissue).

		Isolation						
Test	Specification	#1	#2	#3	#4	#5	#6	#7
Mass of salivary gland tissue	Target mass ≥ 1 g (g)	2.4	0.5	1.8	0.9	1.8	0.8	2.0
Number of total cells after isolation	For information (cells)	1.0 × 10^7^	7.8 × 10^6^	2.1 × 10^7^	7.4 × 10^6^	2.2 × 10^7^	8.1 × 10^6^	9.3 × 10^6^
Presence of clumps after isolation	Present	yes	yes	yes	yes	yes	yes	yes
Bioburden -TAMC -TYMC	<10 CFU/mL <10 CFU/mL	<1/5 mL<1/5 mL	<1/4 mL<1/4 mL	< 1/2mL< 1/2mL	<1/5 mL<1/5 mL	<1/5 mL<1/5 mL	<1/5 mL<1/5 mL	<1/5 mL<1/5 mL
Bacterial endotoxins	<10 EU/ml	<0.5	<0.5	<5.0	<0.5	<5.0	<0.5	<0.5

**Table 8:** The specifications and test results of the technology transfer batches TTR1-6. * Bioburden analysis.

Test	Specification	TTR 1	TTR 2	TTR 3	TTR 4	TTR 5	TTR 6
Passage 0							
Recovery after thawing	For information	88%	111%	94%	94%	89%	57%
Passage 1							
Days in culture from P0	2–4 days	4	4	4	4	4	4
Presence of spheres (before harvest)	Present	present	present	present	Present	Present	present
Number of spheres	for information	1.4 × 10^4^	9.3 × 10^3^	1.5 × 10^4^	1.3 × 10^4^	3.6 × 10^4^	1.7 × 10^4^
Number of cells	for information	8.0 × 10^5^	4.5 × 10^5^	9.0 × 10^5^	1.0 × 10^6^	1.4 × 10^6^	5.7 × 10^5^
Viability	for information	96%	95%	95%	95%	92%	76%
Passage 2							
Days in culture from P1	7–10 days	7	7	7	7	10	10
Presence of spheres (before harvest)	Present	present	present	present	Present	present	present
Number of spheres	for information	6.4 × 10^4^	1.1 × 10^3^	3.0 × 10^4^	5.7 × 10^4^	6.9 × 10^4^	3.4 × 10^4^
Number of cells	for information	2.4 × 10^6^	1.6 × 10^5^	8.4 × 10^5^	7.7 × 10^5^	2.1 × 10^6^	6.9 × 10^5^
Viability	for information	97%	94%	99%	81%	92%	83%
2 days before administration							
Sterility	no growth	0 kve/mL*	0 kve/mL*	no growth	no growth	No growth	No growth
Endotoxins (EU/mL)	<1	<0.5	<0.5	<0.5	<0.5	<0.5	<0.5
Formulation							
Days in culture from P2	7–11 days	8	8	8	8	11	11
Presence of spheres	present	present	present	present	present	present	present
Number of spheres	for information	2.5 × 10^4^	4.7 × 10^3^	1.8 × 10^4^	2.9 × 10^4^	5.5 × 10^4^	2.0 × 10^4^
Number of cells	Target: ≥500,000 cells	6.4 × 10^5^	7.2 × 10^4^	4.6 × 10^5^	5.3 × 10^5^	1.3 × 10^6^	7.4 × 10^5^
Viability	for information	89%	70%	95%	95%	83%	81%
Appearance	Clear to slightly opalescent solution	conform	conform	conform	conform	conform	conform
Sterility	no growth	No growth	No growth	No growth	No growth	No growth	No growth
Endotoxins (EU/mL)	<1	<0.5	<0.5	<0.5	<0.5	<0.5	<0.5

**Table 9:** The specifications and test results of the technology transfer batches TTR 7–TTR 9.

Test	Specification	TTR 7	TTR 8	TTR 9
Passage 0				
Recovery after thawing	For information	77%	95%	87%
Passage 1				
Days in culture from P0	7–10 days	7	7	7
Presence of spheres (before harvest)	Present	present	present	present
Number of spheres	for information	2.2 × 10^4^	9.2 × 10^3^	2.5 × 10^3^
Number of cells	for information	1.7 × 10^6^	1.7 × 10^6^	2.5 × 10^5^
Viability	for information	92%	92%	61%
2 days before administration				
Sterility	no growth	No growth	No growth	No growth
Endotoxins (EU/mL)	<1	<0.5	<0.5	<0.5
Formulation				
Days in culture from P1	7–10 days	9	9	10
Presence of spheres	present	present	present	present
Number of spheres	for information	1.4 × 10^5^	3.0 × 10^4^	3.2 × 10^3^
Number of cells	Target: ≥0.5 × 10^6^	3.3 × 10^6^	1.3 × 10^6^	6.6 × 10^4^
Appearance	Clear to slightly opalescent solution	conform	conform	conform
Viability	for information	97%	93%	50%
Sterility	No growth	No growth	No growth	No growth
Endotoxins (EU/mL)	<1	<0.5	<0.5	<0.5

**Table 10:** The specifications and test results of the technology transfer batches TTR10-11.

Test	Specification	TTR 10	TTR 11
Passage 0			
Recovery after thawing	For information	123%	116%
Days in culture P0	7–10 days	7 days	7 days
Presence of spheres (before harvest)	Present	present	present
Number of spheres	For information	12.2 × 10^3^	25.5 × 10^3^
Number of cells	For information	2.9 × 10^6^	3.6 × 10^6^
Viability	For information	94%	95%
2 days before administration			
Sterility	No growth	No growth	No growth
Endotoxins (EU/mL)	<1	<0.05	<0.05
Passage 1			
Number of cells at start	N.A.	7.2 × 10^5^	7.2 × 10^5^
Days in culture from P1	7–10	10	10
Presence of spheres	Present	present	present
Number of spheres	For information	15.9 × 10^4^	13.0 × 10^4^
Number of cells	≥0.5 × 10^6^ cells	8.0 × 10^6^	5.0 × 10^6^
Appearance	Clear to slightly opalescent solution	conform	conform
Viability	≥70%	98%	97%
Sterility	No growth	No growth	No growth
Endotoxins (EU/mL)	<1	<0.5	<0.5

**Table 11:** The results of histochemical staining of the duct-like structures from the differentiation assay. The cells from TTR 7–TTR 9 were used for the staining experiments.

Batch	Passage	Medium	Days	Alcian Blue	PAS	Aqua-Porin	Amylase	CK19
TTR 7	P0	RYT	30	−	+	−	−	+
TTR 7	P1	RYT	29	+/−	+	−	+	+
TTR 7	P1	WRY	29	+	+	−	+	+
TTR 7	P1	WRY	13	+	+	+	−	+
TTR 8	P0	RYT	30	−	+	−	+/−	+
TTR 8	P2	RYT	30	+	+	−	+	+
TTR 8	P1	RYT	29	−	−	+	−	+
TTR 8	P1	WRY	29	−	+	−	+	+
TTR 8	P2	RYT	13	+	+	−	+	+
TTR 9	P1	RYT	29	+/−	+	−	+	+

**Table 12:** The viability data of the final drug product stored either at room temperature (RT) or at 5 ± 3 °C.

	T = 0	T = 2 h	T = 4 h	T = 5 h
TTR 10/RT	89%	76%	70%	59%
TTR 10/5 °C	87%	80%	75%	71%
TTR 11/RT	84%	75%	60%	54%
TTR 11/5 °C	85%	80%	70%	70%

## Data Availability

Data is contained within the article.

## References

[1] Barsouk A., Aluru J.S., Rawla P., Saginala K., Barsouk A. (2023). Epidemiology, risk factors, and prevention of head and neck squamous cell carcinoma. Med. Sci..

[2] Chow L.Q.M. (2020). Head and neck cancer. NEJM.

[3] Mody M.D., Rocco J.W. (2021). Head and neck cancer. Lancet.

[4] Vissink A., van Luijk P., Langendijk J.A., Coppes R.P. (2015). Current ideas to reduce or salvage radiation damage to salivary glands. Oral. Dis..

[5] do Nascimento M.L., de Farias A.B.S., Carvalho A.T., de Albuquerque R.F., Ribeiro L.N., Leão J.C., Silva I.H.M. (2019). Impact of xerostomia on the quality of life of patients submitted to head and neck radiotherapy. Med. Oral. Patol. Cir. Bucal..

[6] Barazzuol L., Coppes R.P., van Luijk P. (2020). Prevention and treatment of radiotherapy-induced side effects. Mol. Oncol..

[7] Coppes R.P., Zeilstra L.J.W., Kampinga H.H., Konings A.W.T. (2001). Early to late sparing of radiation damage to the parotid gland by adrenergic and muscarinic receptor agonists. Br. J. Cancer.

[8] Vissink A., Mitchell J.B., Baum B.J., Limesand K.H., Jensen S.B., Fox P.C., Elting L.S., Langendijk J.A., Coppes R.P., Reyland M.E. (2010). Clinical management of salivary gland hypofunction and xerostomia in head-and-neck cancer patients: Successes and barriers. Int. J. Radiat. Oncol. Biol. Phys..

[9] Hajiabbas M., D’Agostino C. (2022). Bioengineering in salivary gland regeneration. J. Biomed. Sci..

[10] Silver A.R., Som P.M. (1998). Salivary glands. Radiol. Clin. N. Am..

[11] Mendenhall W.M., Mendenhall C.M., Mendenhall N.P. (2014). Submandibular gland-sparing intensity modulated radiotherapy. Am. J. Clin. Oncol..

[12] Jensen S.B., Vissink A., Limesand K.H., Reyland M.E. (2019). Salivary gland hypofunction and serostomia in head and neck radiation patients. J. Natl. Cancer Inst. Monogr..

[13] Neumann Y., David R., Stiubea-Cohen R., Orbach Y., Aframian D.J., Palmon A. (2012). Long term cryopreservation model of rat salivary gland stem cells for future therapy in irradiated head and neck cancer patients. Tissue Eng. Part C.

[14] Maimets M., Rocchi C., Bron R., Pringle S., Kuipers J., Giepmans B.N., Vries R.G., Clevers H., de Haan G., van Os R. (2016). Long-term in vitro expansion of salivary gland stem cells driven by Wnt Signals. Stem Cell Rep..

[15] Pringle S., Maimets M., van der Zwaag M., Stokman M.A., van Gosliga D., Zwart E., Witjes M.J., de Haan G., van Os R., Coppes R.P. (2016). Human salivary gland stem cells functionally restore radiation damaged salivary glands. Stem Cells.

[16] Rocchi C., Jellema-de Bruin A., Baanstra A., van Rooij N., Brouwer U., van Os R., Jorritsma-Smit A., Barazzuol L., van Zanten J., Coppes R.P. (2021). GMP compliant isolation and expansion of primary human-derived salivary gland organoids for autologous cell-based therapy for xerostomia. Thesis “Exploring the Regereneation Potential of Salivary Glands Using Organoids as a Model”.

[17] Nanduri L.S., Baanstra M., Faber H., Rocchi C., Zwart E., De Haan G., Van Os R., Coppes R.P. (2014). Purification of ex vivo expansion of fuly function salivary gland stem cells. Stem Cell Rep..

[18] European Pharmacopoeia (Ph. Eur.) (2017). General chapter 5.2.12. Raw Materials of Biological Origin for the Production of Cell Based and Gene Therapy Medicinal Products.

[19] Van Luijk P., Pringle S., Deasy J.O., Moiseenko V.V., Faber H., Hovan A., Baanstra M., van der Laan H.P., Kierkels R.G., van der Schaaf A. (2015). Sparing the region of the salivary gland containing stem cells preserves saliva production after radiotherapy for head and neck cancer. Sci. Transl. Med..

[20] Elliott R.L., Jiang X.P. (2019). The adverse effect of gentamicin on cell metabolism in three cultured mammary cell lines: “Are cell culture data skewed?”. PLoS ONE.

[21] Ma Y., Gao L., Tian Y., Chen P., Yang J., Zhang L. (2021). Advanced biomaterials in cell preservation: Hypothermic preservation and cryopreservation. Acta Biomater..

[22] Carlander A.L.F., Gundestrup A.K., Jansson P.M., Follin B., Hoeeg C., Kousholt B.S., Larsen R.T., Jakobsen K.K., Rimborg S., Fischer-Nielsen A. (2024). Mesenchymal stromal/ stem cell therapy improves salivary flow rate in radiation-induced salivary gland hypofunction in preclinical in vivo models: A systematic review and meta-analysis. Stem Cell Reviews and Reports.

[23] Blitzer G.C., Rogus-Pulia N.M., Mattison R.J., Varghese T., Ganz O., Chappell R., Galipeau J., McDowell K.A., Meyers R.O., Glazer T.A. (2023). Marrow-derived autologous stromal cells for the restoration of salivary hypofunction (MARSH): A pilot, first-in-human study of interferon gamma-stimulated marrow mesenchymal stromal cells for treatment of radiation-induced xerostomia. Cytotherapy.

